# Antimicrobial Peptides: Interaction With Model and Biological Membranes and Synergism With Chemical Antibiotics

**DOI:** 10.3389/fchem.2018.00204

**Published:** 2018-06-05

**Authors:** Axel Hollmann, Melina Martinez, Patricia Maturana, Liliana C. Semorile, Paulo C. Maffia

**Affiliations:** ^1^Laboratory of Molecular Microbiology, Institute of Basic and Applied Microbiology, National University of Quilmes, Bernal, Argentina; ^2^Centro de Investigación en Biofísica Aplicada y Alimentos, Consejo Nacional de Investigaciones Científicas y Técnicas and National University of Santiago del Estero, Santiago del Estero, Argentina; ^3^Consejo Nacional de Investigaciones Científicas y Técnicas, Buenos Aires, Argentina

**Keywords:** antimicrobial peptides, peptide-membrane interaction, biophysical tools, synergistic effect, model membranes

## Abstract

Antimicrobial peptides (AMPs) are promising novel antibiotics since they have shown antimicrobial activity against a wide range of bacterial species, including multiresistant bacteria; however, toxicity is the major barrier to convert antimicrobial peptides into active drugs. A profound and proper understanding of the complex interactions between these peptides and biological membranes using biophysical tools and model membranes seems to be a key factor in the race to develop a suitable antimicrobial peptide therapy for clinical use. In the search for such therapy, different combined approaches with conventional antibiotics have been evaluated in recent years and demonstrated to improve the therapeutic potential of AMPs. Some of these approaches have revealed promising additive or synergistic activity between AMPs and chemical antibiotics. This review will give an insight into the possibilities that physicochemical tools can give in the AMPs research and also address the state of the art on the current promising combined therapies between AMPs and conventional antibiotics, which appear to be a plausible future opportunity for AMPs treatment.

## Introduction

Antibiotic resistance is a considerable problem in the population regarding public health and clinical practice. Although there are effective treatments for most infections, the overuse of antibiotics during decades has led to the generation of resistance to commonly used antimicrobials. If this threatening situation continues getting worse, particularly, the ineffectiveness of antibiotics would compromise the success of major surgery and cancer chemotherapy. Besides, the resistance problem is starting to complicate the fight against HIV and malaria. In this context, the progressive decrease in the effectiveness of antibiotics due to the emergence of multi-drug resistant pathogens in addition to the lack of choice of new antimicrobials for treatment emphasizes the need for new classes of drugs and their pharmaceutical forms (Rice, 2003; Boucher et al., 2009; Boto et al., 2018).

Antimicrobial peptides (AMPs) appear as a new strategy for beating infections. These molecules have been found in many species from lowly microorganisms to the human innate immune system. Some natural AMPs are products of millions of years of co-evolution of superior organisms with bacteria and they are part of the first line of immune biological defense. Perhaps the most important feature of these peptides as antibiotics relies on their activity against multi-drug resistant bacteria since they are not as strong as certain conventional antibiotics. Another interesting characteristic of these molecules is the time required to kill bacteria, which is extremely rapid compared to chemical antibiotics (Brogden, 2005; Roversi et al., 2014). The development of resistance is certainly a central issue, as with any new class of antimicrobial therapeutics.

In this regard the development of AMPs resistance has been proposed, but it would be less probable to occur than for classical antibiotics, because of the main target these molecules haves, which is the bacterial cytoplasmic membrane and the consequent need to reconfigure this membrane (Marr et al., 2006). Although some mechanisms of resistance to natural AMPs were described [as for example upregulation of efflux pumps, membrane and cell envelope alterations, proteolytic degradation of the peptides, biofilm formation and lipopolysaccharide (LPS) modification (Segev-Zarko et al., 2018)], with proper concentrations and in combination with antibiotics, synthetic AMPs arise as interesting new antimicrobial agents to fight multiresistant bacteria (Fox, 2013; Riool et al., 2017).

To consider these molecules to be a therapeutic option and overcome clinical setbacks, a worldwide work is done with the aim to understand their mechanisms of action, promote the reduction of cellular toxicity, make them protease resistant, make them more stable and manufacture them on a large scale in a cost-effective manner (Marr et al., 2006; Yount and Yeaman, 2012; Mishra et al., 2017).

AMPs are generally believed to target anionic bacterial membranes, and the first force that drives the initial approach between them is the electrostatic interaction between the positively charged aminoacids and the negatively charged cell surface. The next step is the hydrophobic interactions between the amphipathic domains of the peptide and the membrane phospholipids (Brogden, 2005). The mechanism of action of these peptides is generally drastic if the threshold concentration is reached, and leaves the target organism less able to adapt or develop resistance toward AMPs. The mechanisms by which AMPs can traverse microbial membranes are not common to all peptides and seem to depend on the molecular properties of both, peptide characteristics and lipid membrane composition.

A major research area of growing interest is the possibility to use AMPs in combination with chemical antibiotics, in a synergistic mode of action. These combined therapies appear as a promising approach due to the different mode of action of AMPs compared to commonly used antibiotics. In this regard, it is believed that the permeabilization of the bacterial membrane by AMPs allows the antibiotic to easily enter the bacterial cell at higher concentrations. The future development of new kind of synergistic therapies will require a proper biophysical understanding of the peptide-membrane interactions together with the antibiotic biochemical activity.

## AMPs structure

**Figure 1:**
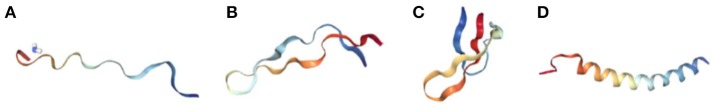
Tertiary structures of representative AMPs. Bovine indolicidin in SDS micelles (PDB ID:1G8C) **(A)**, Bovine lactoferricin (LfcinB) (PDB ID: 1G8C) **(B)**, human α-defensin 4 in an aqueous HEPES buffer (PDB ID: 1ZMM) **(C)**, and human LL-37 in SDS micelles (PDB ID: 2K6O) **(D)**. All-Structures are ribbon diagrams representations obtained from Protein Data bank (PDB; http://www.rcsb.org/pdb/).

We can classify AMPs into four major groups according to their structure: extended AMPs, β-hairpin or loops, β-sheet and amphipathic α-helical (Figure 1) (Powers and Hancock, 2003; McPhee and Hancock, 2005; Aqeel et al., 2012; Gomes et al., 2018).

Extended AMPs are glycine, arginine or hystidine rich peptides that have no secondary structure (Aqeel et al., 2012). Examples include indolicidin (Shaw et al., 2006), histatins (Huo et al., 2011), and drosocin (de Visser, 2005), among others (da Costa et al., 2015).

AMPs that structure as β-sheet are characterized by the presence of two or more β-strands stabilized by disulfide bonds. This class includes α-, β-, and θ-defensins (Corrales-Garcia et al., 2013; Jarczak et al., 2013; Kountouras et al., 2014). β-hairpin AMPs have a hairpin structure interconnected by a type II—turn, and stabilized by disulfide bonds formed between the β-strands, like lactoferricin B, a cationic peptide with a single disulfide bond forming an 18-membered ring between residues Cys2 and Cys20 (Panteleev et al., 2015). Other peptides of this type are tachyplesins and polyphemusins (Laederach et al., 2002; Aqeel et al., 2012).

**Figure 2:**
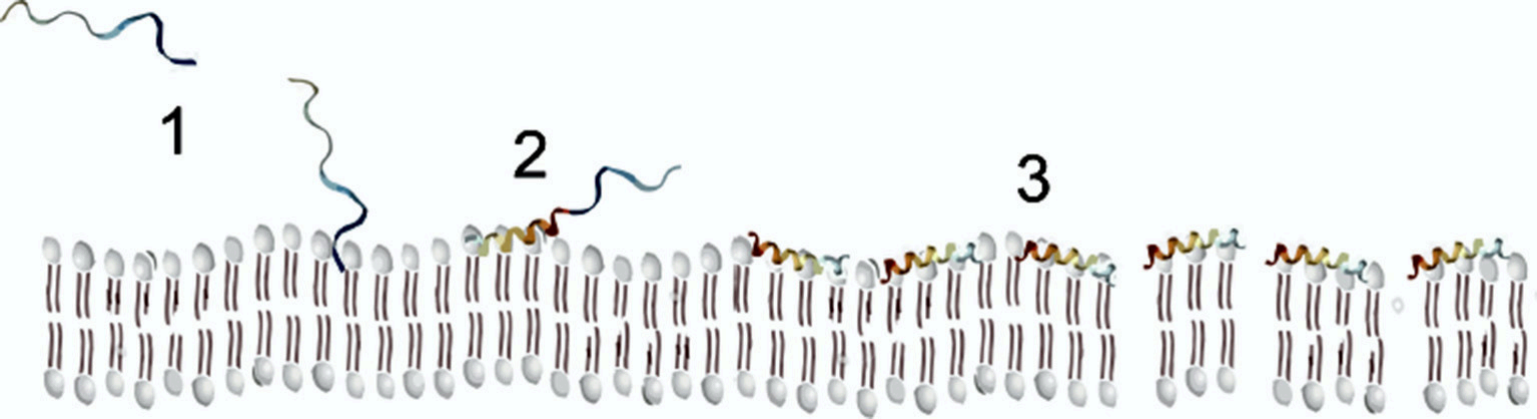
Scheme of AMP membrane interaction following the carpet model.

Finally, α-helical AMPs, due to their amphipathic characteristics, can form α-helical structures in the presence of model or natural membranes (Hollmann et al., 2016). In spite of that, these AMPs are usually unstructured in an aqueous solvent, only in the presence of membranes, they fold into amphiphilic α-helices (Figure 2). When the peptide contacts the negatively charged membrane, it binds to its surface, and above a critical peptide to lipid ratio they can insert into them and form transmembrane pores (Sengupta et al., 2008), provoking a destabilization of the membrane with the subsequent depolarization and cell death. A required concentration threshold is essential for membrane disruption, besides the membrane perturbation model displayed by the peptides (Melo et al., 2009a).

It has been postulated that charged residues within the N-terminal half are important for toroidal pore formation(Mihajlovic and Lazaridis, 2012) and also that peptide aggregation, either prior or after binding to the membrane surface, rather than the helical structure, is a prerequisite to pore formation (Sengupta et al., 2008).

**Figure 3:**
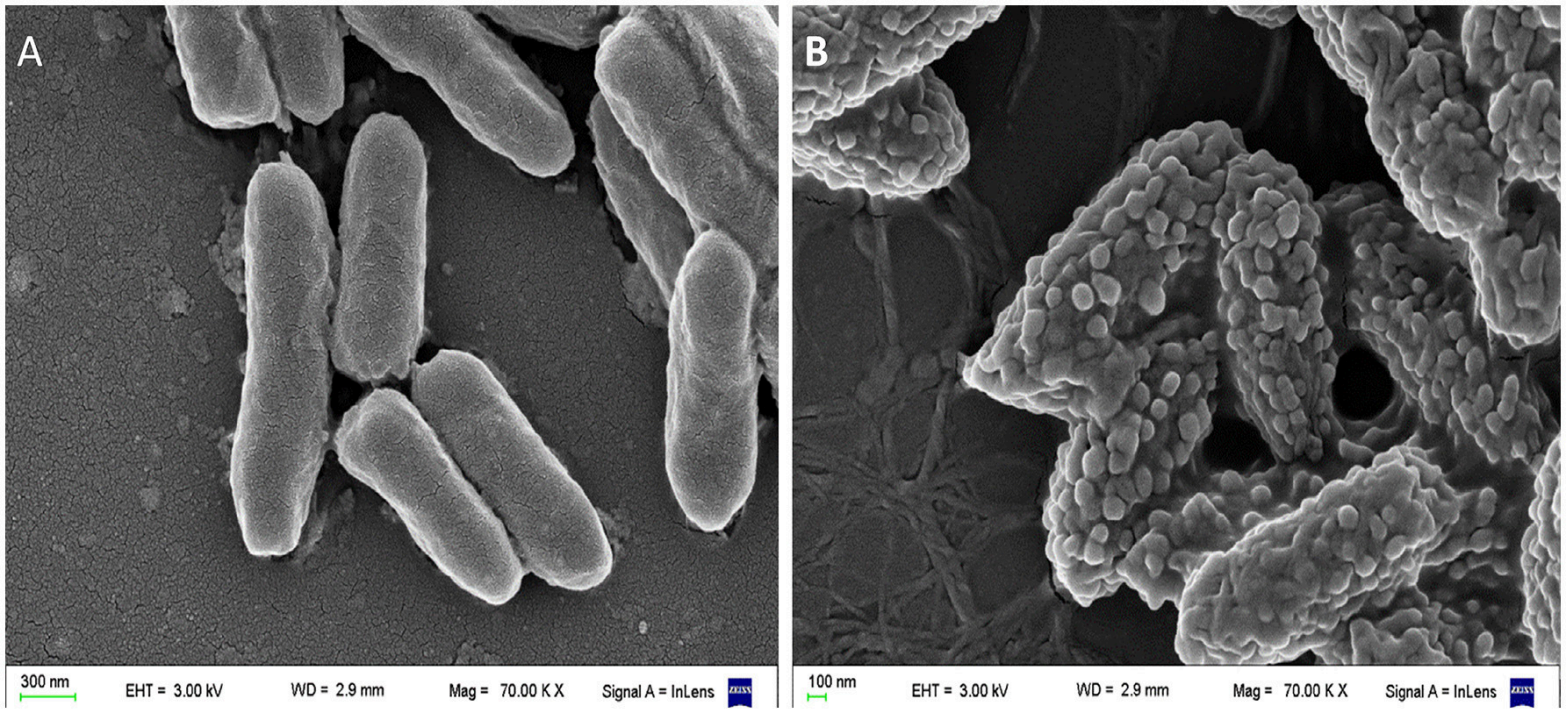
Scanning electron microscopy (SEM) of *Pseudomonas aeruginosa* incubated with a cationic α-helical AMP (P5) designed in our laboratory (Faccone et al., 2014) for 1 h at 37°C at its minimum inhibitory concentration. **(A)** Control: bacteria without treatment. **(B)** Bacteria treated with P5. The latter image shows bacterial cells with the blisters or bubbles protruding from the membrane as a result of the peptide-membrane interaction. Images were taken by our group at the microscopy facility: “Centro de Microscopías Avanzadas”, Facultad de Ciencias Exactas y Naturales, UBA, using a Carl Zeiss NTS SUPRA 40 instrument.

Our scanning electron microscopy (SEM) images of *Pseudomonas aeruginosa* incubated with a cationic amphipatic AMP (Figure 3) show multiple blisters protruding from cell surface; in fact, almost all the bacterial surface was covered with these bubbles after being incubated with P5 [a previously designed cationic amphipathic alpha-helix AMP (Faccone et al., 2014)]. One possible explanation for such phenomenon could be that this kind of AMPs first produce a destabilization of the outer membrane, and afterward disrupt the inner membrane of Gram negative bacteria, so cytoplasm could fill the periplasmic space without disrupting the outer membrane.

## Model lipid membranes

In order to get an insight into the peptide-membrane interactions, physicochemical analysis of AMPs and model membranes are being widely used. In comparison to microbiological assays, biophysical studies on the activity of AMPs strive for a better control of the experimental system at the considerable cost of oversimplifications (Savini et al., 2018).

Physically, model membranes are a simple system, composed of phospholipids, sphingolipids or sterol; but besides its simplicity, model membranes incorporate the most important characteristics of the cell membrane, which is very complex (Nicoli et al., 2010). Generally, these characteristics are relevant to the lateral pressure, lipid composition, and other features that represent the targeted plasma membrane of the living system. Different membrane model systems can be used to understand AMPs interactions at a molecular level. As one of the main components of cellular membrane, phospholipids have excellent biocompatibility. Additionally, their amphiphilic structure confers phospholipids with self-assembly, emulsifying and wetting characteristics (Li et al., 2015).

**Figure 4:**
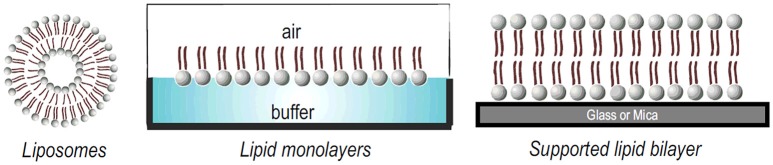
Different model membrane systems used to study lipid-peptide interactions.

Model membrane systems offer an alternative way to the study of membrane—peptide interactions, with the advantage of easily control the conditions to be tested. The primary structure of any membrane is the lipid bilayer. Several model systems have been developed to mimic the properties of this bilayer. Typical examples of model membrane systems are Langmuir monolayers, vesicles or liposomes and solid supported bilayers (Figure 4). All of these systems offer advantages and disadvantages for the study of peptide-membrane interactions (Knobloch J. J et al., 2015).

A liposome is a phospholipid bilayer (membrane) formed into an enclosed pocket. Depending on the mode of preparation, lipid vesicles can range in size from tens of nanometers, like small unilamellar vesicles (SUVs), to 1,000 nm, like large unilamellar vesicles (LUVs) or giant unilamellar vesicles (GUVs) that range up to tens of microns. Liposomes can also be composed by multilamellar vesicles, a structure similar to a matryoshka doll of concentric membranes, known as a multilamellar vesicle (MLV) (Bunker et al., 2016; Jorgensen et al., 2017). For a recent review about their preparation modes, the reader can refer to (Laederach et al., 2002).

Lipid monolayers, also called “Langmuir monolayers” (Figure 4) are single monomolecular layers of a given surface active molecule at the air/liquid interface. The formation of this kind of model membrane is rather simple and consist on deposition of the lipids dissolved in organic solvents onto the liquid surface and, after solvent evaporation, the polar head groups of the lipids stay in contact with the liquid subphase and the non-polar hydrocarbon chains point to the air (Bohinc et al., 2014). This membrane model makes it possible to assess the effect of the peptide on the phase transition of the lipid film, providing information on the changes of lipid packing induced by the peptides. Experiments with monolayers have the unique advantage that the arrangement and packing of the lipid molecules can be easily measured and modulate and it is comparably much easier to control the composition and density of the lipid layer (Knobloch J. et al., 2015).

Finally, supported lipid bilayers (SLBs) (Figure 4) is another interesting model, constituted of a single bilayer supported on a solid substrate, as silica, glass, or mica. The hydrophilic head groups of the lipid face the substrate, separated by a thin hydration layer. SLBs are particularly valuable models due to their lipid arrangement and because they can be easily formed (Hardy et al., 2013). In addition, because they are confined in two dimensions, this planar configuration allows the use of many quantitative surface characterization techniques. These systems typically allow the investigation of interactions with lipid head groups but also can resemble the membrane diffusivity of cells (Hardy et al., 2013). Finally, as SLBs are confined to the surface of a solid support, they can be characterized much more readily than free-floating vesicles using a large variety of surface sensitive techniques such as AFM, quartz crystal microbalance (QCM), infrared reflective absorption spectroscopy (IRRAS) SPR, among others (Mingeot-Leclercq et al., 2008; Goksu et al., 2009; Brand et al., 2018).

Another advantage of working with lipid membrane is related to the feasibility of modulating its lipid composition in order to mimic different kind of membranes. The selectivity of many peptides has been investigated using negatively charged vesicles that mimic bacterial membranes or neutral (or zwitterionic) vesicles that mimic eukaryotic membranes. These latter model membranes have also been used to assess the mechanisms implied on the hemolytic activity. For instance, phosphatidyl choline (PC) is usually employed to mimic the membrane of mammalian cells, as PC is one of the major component of its cytoplasmic membranes, whereas phosphatidyl glycerol (PG) is usually added on bacterial-like model membranes, since it is absent in the outer leaflet of eukaryotic plasma membranes, but is abundant in bacterial membranes (Shireen et al., 2015). A lot of physicochemical techniques and membrane models have been applied to AMPs characterization during the last decades (Vestergaard et al., 2017). However, it should be pointed that the power of using model membrane systems is revealed when different model architectures are used to study peptide-membrane interactions of the same peptide (Savini et al., 2018).

## AMPs membrane interaction

### Affinity and partition

Besides the chosen membrane perturbation model, membrane disruption is achieved only after the AMP reaches a threshold concentration in the membrane, as supported by several observations in model systems (Melo et al., 2009b). To understand the AMPs mechanism of action, knowledge of the binding affinity of these molecules to potential cellular binding sites is essential. In this context there are two major questions in order to characterize peptide-lipid interactions: (1). how many peptides reach the membrane? and (2). with what affinity? Regardless of the techniques used to measure the binding reaction (i.e., kinetic approaches, equilibrium methods), the lipid binding affinity and amount of peptide bound to the membrane can be easily described by two different thermodynamic models *partition equilibrium* and *binding affinity*.

### Partition equilibrium

A usual biophysical approach for studying the interaction between AMPs and membranes involves the determination of the extent of peptide partitioning or binding to model membranes, which is commonly translated into partition constants. It is usually accepted that the partition constant, *K*_*p*_, is defined as the concentration ratio of the peptide between the lipidic and aqueous phases.

For the determination of the partition coefficient has been achieved by using different methodologies, such as UV-Vis absorption spectrophotometry, fluorescence spectroscopy and Z-potential measurements (Freire et al., 2011). For a detailed information about the different techniques and equations used to obtain partition coefficients with spectroscopic techniques, the reader can refer to Santos et al. (2003). Partition calculation without physical separation of the “lipid phase” from “aqueous phase” is the major advantage of these techniques in comparison to chromatographic techniques (Matos et al., 2010; Ribeiro et al., 2010).

Using these approaches, it has been possible to determine the fraction of the total peptide concentration that is actually bound to the lipid bilayers. Coupling these data to the peptide-induced leakage experiments, it is easy to obtain the threshold concentration of peptide (per lipid or per vesicle) that is needed to cause membrane perturbation.

For example, Bouchet et al. (2014) reported, for the AMP [K^108^W^111^] 107–115 hLz, a *K*_*p*_ of 3.2 × 10^3^ for pure DMPC, whereas this value rises to 17.3 × 10^3^ on PG containing membranes. Thereby, working in a high molar lipid excess condition (3 mM), we obtain an *X*_*L*_(molar fraction of the peptide on the membrane) of 0.88 on pure DMPC and when we move to negatively charged membranes that value increases to 0.97 (i.e., almost all peptides added keep bound to the membrane in this condition).

AMP selectivity and safety is partially explained by the fact that these partition constants are usually significantly higher for bacterial model membranes than mammalian ones, and is considered in the rational design of new peptides. However, the analysis of partition constants has usually been limited to this biophysical approach. Partition constants are rarely considered in an absolute sense, but are usually used to compare different systems (Savini et al., 2017).

### Binding affinity

This chemical binding model, assume that the peptide (P) have *n* equivalent and independent binding sites for substrate [i.e., the lipid (L)], according to the reaction: P +n L⇄PLn, where the bound lipid over total peptide concentration could be expressed as *k*_*on*_[*L*][*P*] = *k*_*off*_[*PLn*], where *k*_on_ and *k*_off_ are the association and dissociation rate constants. From this assumption it could be obtained the apparent dissociation constant (*K*_*d*_) expressed as a relation between *k*_*off*_ and *k*_*on*_ constants (Hulme and Trevethick, 2010). Therefore by testing different lipid compositions we can have information about the lipid selectivity of the peptides. Many spectroscopic methods, based on the binding perturbation of the electronic and spectroscopic energy levels of the membrane or peptide can be used in order obtain affinity information, as infrared (IR), UV–visible, fluorescence (Tiriveedhi et al., 2011), optical rotatory dispersion (ORD), nuclear magnetic resonance (NMR), Plasmon Waveguide Resonance (PWR) (Jobin et al., 2013), circular dichroism (CD), and Dynamic Light Scattering (DLS) (Faustino et al., 2014). These methods can be performed in solution, which is a great advantage as it enables true equilibrium measurements (for a detailed review of the advantages and disadvantages of each techniques the reader can refer to Vuignier et al., 2010).

Both parameters, partition coefficient and dissociation constant, are widely used to characterize the interactions of AMPs with different model membranes and several lipid compositions in order to predict antimicrobial or cytotoxic activities (Table 1). Recently, by using different model membranes made from different lipid compositions, we have come to the conclusion that the selectivity of the peptide between zwitterionic or negatively charged lipids determines its bioactivity (Maturana et al., 2017); in other words, affinity toward negatively charged lipids instead of zwitterionic ones seems to be a determinant feature that drives from hemolytic to antimicrobial results.

**Table 1:** Partition coefficient, *Kp*, and dissociation constant, *K*_*d*_, for *de novo* synthetics AMPs 5, 8, and 8.1 (Hollmann et al., 2016).

**Peptide**	**Lipid composition**	***K_*p*_***	***I*_max/Iw_**	***K_*d*_***	**Π_max_**
pep5	DMPC	2, 154 ± 417	1.95 ± 0.05	1.06 ± 0.20	11.95 ± 0.83
	DMPC:DMPG (5:1)	16, 281 ± 1, 692	1.82 ± 0.02	0.30 ± 0.07	11.90 ± 0.50
pep8	DMPC	37, 684±11, 138	2.00 ± 0.01	0.40 ± 0.15	13.65 ± 0.93
	DMPC:DMPG (5:1)	9, 696 ± 738	2.37 ± 0.02	0.18 ± 0.07	13.64 ± 0.65
pep8.1	DMPC	−	−	0.71 ± 0.22	6.91 ± 0.63
	DMPC:DMPG (5:1)	5, 233 ± 641	1.86 ± 0.02	0.15 ± 0.13	12.17 ± 1.11

### Final position and structure into the membrane

**Figure 5:**
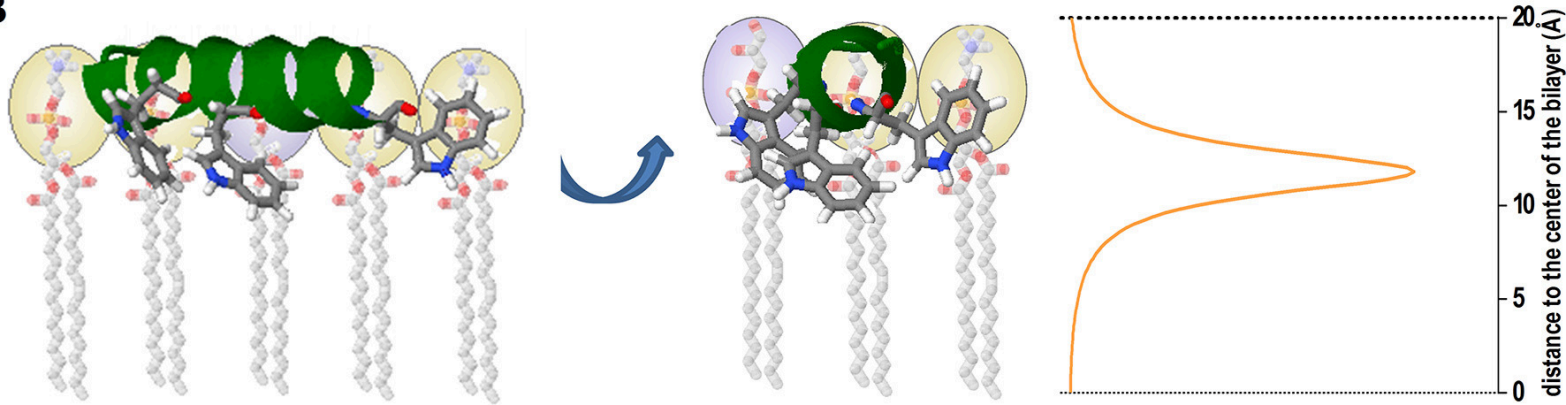
Cartoon representation depicting an example of the use of the SIMEXDA method (right plot) to visualize the in-depth localization of Trps residues of an alpha-helix structured cAMP inside the membrane. Reprinted with permission from Maturana et al. (2017) © Colloids and Surfaces B, Biointerfaces, Elsevier.

The partition coefficient or affinity constant does not contain, in principle, information about the solute location (i.e., the peptide) in the membrane, which can be either adsorbed at the membrane interface or internalized at different depth into the lipid bilayer. This structural information can be obtained by using other spectroscopic methodologies such as differential fluorescence quenching (Nicoli et al., 2010; Knobloch J. J et al., 2015; Li et al., 2015). In this technique quencher molecules that take different positions in the membrane are used, as 5NS and 16NS Stearic acid molecules derivatized with doxyl (quencher) groups either at carbon 5 or 16, respectively. 16NS is a better quencher for molecules buried deeply in the membrane, while 5NS is better for molecules inserted in the membrane in a shallow position, close to the lipid-water interface (Fernandes et al., 2002). As molecular contact is required between the quencher and the fluorophore (i.e., Trp residues of an AMP) the efficiency of quenching is related to the proximity between quencher and fluorophore and allows us to calculate their final position in the membrane (Figure 5). Quenching experiments also allow to determine the final position of AMPs from different origins, as well as to contribute to unraveling the mode of action of these peptides (Bouchet et al., 2014; Hollmann et al., 2016; Maturana et al., 2017).

### Membrane disruption

As was pointed above, induced leakage of bacterial content is perhaps the most common killing mechanisms of peptides with antimicrobial, host defense or cytotoxic functions. In this context, membrane permeabilization studies in model membranes are a key factor in the characterization of AMPs with lipid bilayers.

The membrane leakage fluorescence assay based on vesicles loaded with self-quenching dyes has been widely used for quantifying the activity of antimicrobial peptides that can permeabilize membranes and produce leakage of the entrapped contents. This experimental technique makes use of the self-quenching properties of the dye, because it is entrapped at high concentration. The release of the dye from the vesicles to the media implies a dilution of the dye that can be monitored directly by increasing fluorescence intensity. Many different fluorescent dyes and quenchers, such as a pair 8-aminonapthalene-1,3,6 trisulfonic acid (ANTS)/p-xylene-bis-pyridinium bromide (DPX), that change fluorescence intensity upon membrane leakage can be used (Kyrychenko, 2015). Calcein and Carboxyfluorescein are also fluorescent probes widely used in leakage from liposomes to measure membrane permeabilization caused by AMPs. The level of permeabilization was shown to depend on the lipid composition and, therefore, on the physical properties of the bilayers (Knobloch J. et al., 2015). This LUV suspension method only provides an average of the physical properties such as fluorescence intensity among these LUVs in different stages of the reaction. It is therefore difficult to elucidate the detailed information about the disruption mechanisms involved. For example, if half of the encapsulated solute has escaped, this can either be the result of half of the vesicles releasing all their content, or all vesicles releasing half of their content. In both cases we will detect a 50% leakage, and we will not elucidate if it is due to the result of an all-or-none or a graded mechanism (van Rooijen et al., 2010). In summary, the LUV suspension method provides information about some AMP-induced leakage, but the elementary processes of the leakage and the mechanistic details of the interactions remain unclear (Islam et al., 2014). In contrast, the mechanism of the disruption process on model membranes can also be achieved by confocal fluorescence microscopy using giant unilamellar vesicles (GUVs), particularly the single GUV method allows to observe the interaction of an AMP with a single GUV and the induced leakage of a fluorescent probe from the inside of the single GUV as a function of time using fluorescence microscopy. van Rooijen et al. (2010) used POPG GUVs encapsulated with the dye 8-hydroxypyrene-1,3,6-trisulfonic acid (HPTS) and a rhodamine-labeled lipid was added to visualize the lipid bilayer. To further reduce the signal from un-encapsulated HPTS, the quencher p-Xylene-bis, N-pyridinium bromide (DPX) was added to the solution outside the GUVs. When oligomeric α-Synuclein was added to the imaging chamber, the fluorescence from the vesicle interior was lost, either through HPTS efflux or DPX influx. Using the single GUV method, Tamba and co-workers (Tamba and Yamazaki, 2009), found that the AMP magainin-2 induce pore formation in the membrane but it greatly depended on the surface charge density of the membrane and the salt concentration in the buffer.

### *In silico* prediction of membrane-peptide interactions

A number of theoretical and computer simulation approaches have been developed to describe bioactive molecule/lipid interactions. The different approaches vary in the way the system is modeled, and therefore in the kind of information that each particular model can give us (Deleu et al., 2014) One of the approaches to characterize “*in silico*” interaction between peptides and membranes is molecular dynamics (MD), in which the dynamics of a molecule into the lipid bilayer and its effects on surrounding lipids are investigated. One of the main advantages of MD simulations is the ability to describe, at atomic resolution, the probable location and orientation of drugs into the membrane. These parameters have been successfully predicted for a wide variety of drugs, in a good agreement with experimental data obtained on membrane model systems (Di Meo et al., 2016). Recently, Berglund et al. (2015) used MD in order to characterize the interaction of Polymyxin B1 (PMB1), a well-known small antimicrobial lipopeptide, with a heterogeneous model of the bacterial outer membrane of *Escherichia coli*, revealing the contrasting behavior of PMB1 in the presence of different bacterial membrane models. This lipopeptide aggregates in the lipopolysaccharide headgroup region of the outer membrane, showing a limited tendency for insertion within the lipid A tails; however, on the other hand, it readily insert into the inner membrane core, increasing lipids hydration which is responsible for bilayer destabilization and antimicrobial function.

Sudheendra et al. (2015) studied different analogous of human β-defensin-3 (HβD-3) by many different techniques. Using MD of POPC bilayers, modeled with the peptides embedded within the bilayer and solvated with a 10Å water box, they found that with the course of the simulation the terminal residues were protruding out of the micelle environment. They reach to the conclusion that membrane disintegration is governed by electrostatic interactions, where the electropositive charge of terminal residues of the peptide disrupts the phosphate head group and accordingly the peptide gets distended out.

Another “*in silico*” methodology is the molecular docking, a reliable tool able to locate the probable binding interactions of ligands with their target proteins (Bhosale et al., 2018). This technique is suitable for investigations related with molecular events, including ligand binding modes and the corresponding intermolecular interactions that stabilize the ligand-receptor complex.

Molecular docking programs through a cyclical evaluated ligand conformation by specific scoring functions until converging to a solution of minimum energy (Huang et al., 2010; Mulholland et al., 2016) have recently studied by docking the nisin2:lipid II complex in bacterial membranes, which has been put forward as the building block of nisin/lipid II binary membrane pores. The authors used AutoDock Vina, wich, like many docking programs, produces a binding affinity that is useful for comparing different conformations and ligands (Trott and Olson, 2010). Docking methods are particularly useful to screen specific interaction of peptides with different lipids and hence to understand its membrane activity and specificity. For example, Fantini et al (Fantini et al., 2011) using docking studies revealed that the 69–79 fragment of α-synuclein displays a potential binding site for cholesterol. Nsimba Zakanda et al. (2012) by applying a Hypermatrix method compared the affinity of hexadecylbetainate chloride (C16BC) with POPC, sphingomyelin and cholesterol, three biological relevant lipids present in the outer leaflet of the mammalian plasma membrane. Finally, Deleu et al. (2014) improving the HM docking approach, calculated the interaction between a lipopeptide (as surfactin) with different lipids as DPPC, DOPC and binary mixtures of them. This method allows observing the preferential interactions and phase separation between the molecules under consideration.

## Interactions with biological membranes

Biophysical techniques on model membranes can provide very detailed information on the interaction of AMPs with membranes, and most of our knowledge of the molecular details of the way of action of the peptides comes from this kind of studies. However, until recently, it was unknown if any of the models proposed, such as the “carpet” mechanism, were relevant for AMPs activity in real bacteria (Savini et al., 2018). In this context, in recent years, some techniques typically applied in model membranes started to be applied to bacteria, like Zeta Potential and some fluorescent approaches as dipole potential perturbations.

Alves et al. (2010) showed a correlation between antimicrobial susceptibility and bacterial surface charge neutralization assessed by zeta potential, using the antimicrobial peptide BP100 or pepR on *E. coli*. Furthermore, some insights into the mode of action of AMPs against *E. coli*, at an atomic level, were achieved by whole-cell AFM imaging. Avitable and co-workers (Malgieri et al., 2015) explored the interaction of Gram negative bacteria cells with antimicrobial peptides. In this work, the authors used CD to gain information on the secondary structure the peptides assume once they meet the bacterial cells and demonstrated that CD is a technique suitable for studying the interaction of peptides with *E. coli* cells. In another work by Roversi and co-workers, the association of an analog of the AMP PMAP-23 to *E. coli* cells was determined and found that killing took place only when bound peptides completely saturated bacterial membranes (10^6^–10^7^ bound peptides per cell). These results lead the authors to conclude that the “carpet” model for the perturbation of artificial bilayers is representative of what happens in real bacteria (Roversi et al., 2014).

Interestingly, in all these studies, among others, similarities between model membrane-based and bacteria-based work were found (Freire et al., 2011). These findings support the concept that model membranes, within reasonable limits, still represent a reliable model to characterize AMPs.

One issue to be considered is selectivity, since liposome studies usually indicate that AMPs have different affinities for bilayers mimicking the membranes of bacteria or eukaryotes. Besides, it has usually been claimed that the difference in lipid composition of membranes of the two cell types could define a higher affinity for bilayers mimicking bacterial membranes. However it might be just an experimental artifact resulting from the very different conditions used in the assays used to determine antimicrobial and hemolytic activities (Bobone et al., 2013), because direct peptide affinity toward the two types of cells has been poorly explored (Savini et al., 2018). In this context, we have recently shown that dipole potential assays could be directly applied to erythrocytes, giving us information about peptides affinity with this kind of cell membranes. Moreover, these affinities match perfectly with the hemolysis results (Hollmann et al., 2016; Maturana et al., 2017). Nevertheless, the use of separate assays is based on the assumption that the affinity of AMPs against a given cell type is not influenced by the contemporary presence of different cells. Savini et al. (2017) also evaluated the bactericidal effect of a fluorescently labeled analog of the cathelicidin HDP PMAP-2315 in the presence of RBCs. Surprisingly, in these assays, the antimicrobial activity was not affected by the presence of RBCs, and peptide toxicity remained the same in the absence or presence of the bacteria. Furthermore, in the same work, killing and hemolysis curves for the AMP esculentin-1a(1–21)NH2 were measured in the presence of both bacteria and erythrocytes or in the presence of only one cell type and just a minor inhibition of the antibacterial activity was caused by the presence of a large excess of RBCs. It is worth to notice that esculentin-1a(1–21)NH2 is an AMP derived from esculentin-1a, a peptide with a high therapeutic index (77 for *E. coli*) (Islas-Rodriguez et al., 2009).

## Cytotoxicity vs. antimicrobial activity

A crucial factor for AMPs activity is determined by the phospholipid composition and the net charge of the target cell membranes (Yeaman and Yount, 2003). In fact, the mammalian cell toxicity is a possible undesirable property, particularly, the erythrocytes selectivity represents a challenge to be taken into account when designing new AMPs (Melo et al., 2009a)

The selectivity of an AMP toward eukaryotic or prokaryotic membrane is often measured by an index called therapeutic index, which is defined as the ratio between their minimum hemolytic (MHC) and minimum inhibitory concentrations (MIC): (Jiang et al., 2008; Melo et al., 2009a). The therapeutic index reflects the effectiveness that the AMP would have as an antibiotic, the higher the value of the therapeutic index, the better performance of the AMP as an antibiotic (Zelezetsky and Tossi, 2006).

Some researchers have tried to develop novel AMPs with low toxicity and improved antimicrobial activity (Hawrani et al., 2008). Accordingly, there have been many attempts to clarify the parameters that control the selectivity of AMPs (Kim et al., 2014).

The importance of the amphipathicity on the antimicrobial or hemolytic activity has been debated. In some works (Jiang et al., 2008) it has been stated that net charge instead of increasing amphipathicity is the key parameter to explain biological activity; however, we and others have shown that amphipathicity is a requirement for cationic AMPs activity when these peptides get an α-helix structure (Takahashi et al., 2010). In any case, perfect amphipathicity produce a simultaneous increase in the hemolysis and bactericidal activity. In our previous work, in order to get an insight into the possible factors involved in the type of lipid membrane selection by cationic AMPs, we designed two model peptides from a previously reported AMP (Hollmann et al., 2016; Maturana et al., 2017). These new sequences displayed very different activities on biological membranes. One of the resulting AMP displayed a continuous hydrophobic face and a disrupted hydrophilic face, this feature prevented the peptide from having antibacterial activity, but prompted it to have high hemolytic activity. For the second peptide, after some selected aminoacids substitutions, we simply turned it amphipathic and in turn it became active against bacterial membranes and considerably diminished its hemolytic activity (Maturana et al., 2017).

We could see that an increase in net charge on the polar face of an amphipathic α-helix AMP also increases its hemolytic and antimicrobial activity.

Several authors demonstrated the relevance of peptide hydrophobicity in membrane selectivity and insertion, besides the antimicrobial activity (Dathe et al., 1997; Chen et al., 2007). It has also been postulated that cationic AMPs with increased hydrophobicity can penetrate zwitterionic membranes and provoke hemolysis. Furthermore, at low concentrations, peptides with the same hydrophobicity display a reduced hemolysis if they featured a separate distribution of positive charges (Yin et al., 2012).

Working with related AMPs we could determine that highly amphipathic α-helical AMPs with a high hydrophobic moment are probably responsible for eukaryotic membrane affinity, hence producing high hemolytic activity (Faccone et al., 2014).

It has also been postulated that disarranging perfect amphipathicity of a α-helix peptide can sustain its antimicrobial activity and induce pore formation, while reducing hemolytic activity (Mihajlovic and Lazaridis, 2012).

Helicity is another crucial parameter for the biological activity of α-helical antimicrobial peptides (Huang et al., 2012). We have previously shown that, at least for the sequences studied, helicity promotes the biological activity of a group of *de novo* designed α-helical AMPs (Faccone et al., 2014). Our results are in agreement with other works (Huang et al., 2014) that demonstrated that peptide helicity displays a critical role in the antimicrobial activity of α-helical antimicrobial peptides.

## Synergistic activity with other antibiotics

We can say that two compounds are synergistic when its combinations can exert inhibitory effects that are more than the sum of their effects alone. The rationale for AMP-antibiotics combined therapies relies on the fact that the mode of action of membrane permeabilizing is extremely different from conventional chemical antibiotics. As we have mentioned before, the major mechanism of action of AMPs is conducted over bacterial membranes, producing membrane destabilization or pore formation, followed by loose of osmotic balance and, finally, cell lysis. It has been proposed that this feature probably facilitates the entrance of other conventional antibiotics when AMPs are used in combination with such drugs, promoting synergistic activity that may affect different targets inside the bacterial cell.

**Figure 6:**
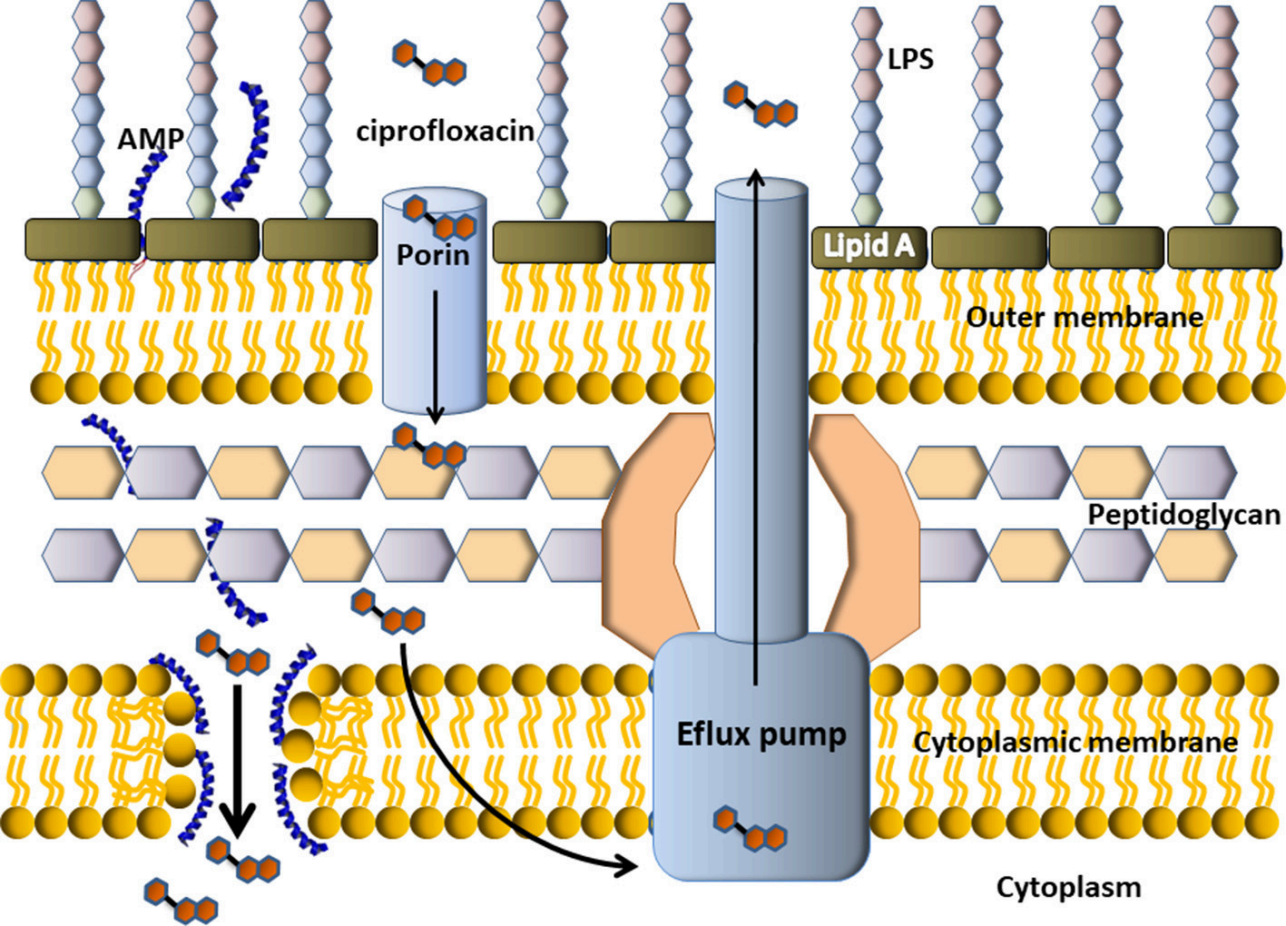
Scheme of putative synergism between a cationic α-helix AMP with a conventional antibiotic. Cefalosporin is a DNA gyrase inhibitor, so it must get inside the bacterial cell to display antimicrobial activity. In the example, the resistance mechanism of this Gram-negative bacteria is an efflux pump, which pumps the antibiotic outside the cell. The AMP would permeabilize the membrane thus producing the income of more antibiotic molecules to the cytoplasm, where they could finally get the target.

Conventional chemical antibiotics display antimicrobial activity, in general, inhibiting DNA replication, DNA transcription or cell-wall synthesis, particularly, targeting topoisomerases and penicillin-binding proteins (PBPs). Some of the drug resistant mechanisms displayed by multidrug resistant (MDR) bacteria result in the difficulty of these molecules to enter the bacterial cell, which leads to the decrease of titre. On the other hand, AMPs target the cytoplasmic membrane and induced an increased permeability and a loss of barrier function. The disassembled bacterial membrane is now permeable to chemical antibiotics, that can enter the cytoplasm and attack their targets. Figure 6 depicts an example of a possible mechanism (but certainly not the only one) involved in the synergistic effect seen for the combination of some AMPs with chemical antibiotics. In this example, the cartoon representation shows an efflux pump-mediated ciprofloxacin resistance in Gram-negative bacteria. The AMP permeabilizes the membrane so more antibiotic can enter the bacterial cell and get the target. In this example, the targets of ciprofloxacin are the enzymes topoisomerase II (DNA gyrase) and topoisomerase IV (not shown).

The use of combined therapy must be balanced against possible disadvantages as antagonism, superinfection, increased incidence of adverse effects and increased cost. In clinical practice, it is widely used in life-threatening infections, with the aim of reaching all potential pathogens when a single antimicrobial agent has not a broad spectrum of action. Sometimes, the choice of combined therapy is done on purpose to avoid resistance emergence or when a polymicrobial infection not treatable with a single drug exists (Bouza and Munoz, 2000).

Some AMPs have been evaluated in combination with conventional antibiotics or with other AMPs and synergistic activity was demonstrated against a wide range of very important human pathogens.

Colistin, a cationic antimicrobial peptide, synergizes with azithromycin, erythromycin and clarithromycin against MDR *K. pneumoniae, P. aeruginosa* and *A. Baumannii* (Lin et al., 2015). It has been proposed that this AMP permeabilizes the bacterial membrane thus facilitating the entrance of the antibiotic, whereupon it can exert its classical ribosomal protein synthesis inhibition activity. They also demonstrated marked synergy between azithromycin and LL-37, a human cationic AMP produced during infectious processes, against the same three MDR-Gram negative rods.

Daptomycin is a cyclic lipopeptide antibiotic, its mode of action includes the association with calcium to form a cationic complex and is active against Gram-positive organisms. Sakoulas et al. reported a case of aortic valve endocarditis caused by vancomycin- and ampicillin-resistant *E. faecium* (VRE), in a patient with bacteremia refractory to therapy with daptomycin. A combined therapy of daptomycin and ampicillin was employed for this patient, based on prior *in vitro* studies showing synergy between these two antimicrobials. Interestingly, in 24 h, the persistent bacteremia was cleared with the combined regimen. Moreover, they investigated the effects of ampicillin on the VRE strain and demonstrated that exposure to ampicillin induced a reduction in the net positive charge of the surface that was associated with greater surface binding of daptomycin. (Sakoulas et al., 2012).

Human β-defensin 3 (HBD3) and the cathelicidin LL-37 exhibited synergistic effects in combination with tigecycline, moxifloxacin, piperacillin-tazobactam and meropenem, on *Clostridium difficile* strains, even against those strains that were mostly resistant to moxifloxacin and meropenem, as demonstrated by Sabine Nuding and co-workers (Nuding et al., 2014). They found that the degree of synergism between AMPs and antibiotics was strain dependent and that membrane disturbance caused by AMPs may increase the antibiotic uptake promoting the antibacterial effect of the combination therapy.

In light of this, it has been proposed that minimal inhibitory concentration (MIC) testing disregards potential synergies between antimicrobials and other AMPs (host AMPs included), that promote bactericidal activity *in vitro* and bacterial clearance in patients (Sakoulas et al., 2012; Lin et al., 2015).

Additionally, nowadays other strategies involving peptide antimicrobial activity are being studied. Recently, some peptidomimetic compounds have been reported, as for example, ultrashort antimicrobial peptidomimetics based on lysine that is comprised of aromatic and aliphatic alkyl groups directly appended to the C-terminus of L-lysine. These molecules, denominated amphiphilic lysines, have broad-spectrum antimicrobial activity against Gram-positive and Gram-negative pathogenic bacteria (Zuckermann and Kodadek, 2009; Ghosh et al., 2014). Furthermore, a hybrid prepared by covalently fusing an ultrashort amphiphilic lysine with the aminoglycoside tobramycin has reported to potentiate the therapeutic utility of these peptidomimetics. The conjugate has augmented activity respect the amphiphilic lysine alone against *P. aeruginosa PAO1*, and significantly synergizes 8 clinically used antibiotics against *P. aeruginosa PAO1* and MDR/XDR *P. aeruginosa* clinical isolates (Lyu et al., 2017).

Feng et al. (2015) demonstrated synergistic activity between a group of short cationic antimicrobial peptides combined with traditional antibiotics against Gram-negative bacteria (*Escherichia coli, Klebsiella pneumoniae*, and *P. aeruginosa*), Gram-positive *(Staphylococcus epidermidis, Streptococcus pneumoniae*, and *Staphylococcus aureus*,) Some of the peptides evaluated were from the laboratory library, some were analogs of a melittin B and cecropin A hybrid peptide, and others were derived from the N-terminus of L1, the ribosomal protein of *Helicobacter pylori*. Almost all peptides exhibited a synergistic effect in combination with cefepime, imipenem, vancomycin, and levofloxacin hydrochloride *in vitro*. Additionally, in this study, synergy was evaluated *in vivo* in a mouse wound infection model. Combined therapy with PL-5 and levofloxacin hydrochloride significantly decreased the CFU number of *Staphylococcus aureus* at the higher dose evaluated, demonstrating synergistic effects *in vivo*.

Ocellatin peptides, a family of cationic antimicrobial peptides isolated from skin secretion of the frog *Leptodactylus pustulatus*, exhibited good antimicrobial activity against *P. aeruginosa* (Bessa et al., 2018). Interestingly, this AMP showed higher antimicrobial activity in MDR isolates than in the susceptible strains assayed. It might be due to the loss of natural impermeability to ocellatins of the multi-resistant strains. Ocellatins were also evaluated in combination with antibiotics with different mechanisms of action and the results showed increased antimicrobial activity. Particularly, ocellatin-PT3 in combination with ceftazidime or ciprofloxacin showed the best synergistic effect against *P. aeruginosa* Pa4-SA2. Moreover, Ocellatin-PT3 inhibited mature biofilm proliferation in concentrations up to 10xMIC, while ciprofloxacin could not produce inhibition effects even in 32xMIC concentration.

The cationic peptide nicin is a 3.5 KDa lantibiotic (a type of AMPs that contain lanthionine or methyllanthionine) produced by *Lactococcus lactis*. Lewies et al. (2017) studied the interaction of nisin Z, a naturally occurring variant of nisin, with conventional antibiotics on *Staphylococcus aureus, Staphylococcus epidermidis* and *E. coli*. They found that nisin Z exhibited additive interactions with conventional antibiotics and a remarkable synergism with novobiocin.

Although there are several examples of synergistic combinations between AMPs and conventional antibiotics in recent literature, not every combination between AMPs and chemical antibiotics results in synergism. The radically different mode of action between these membrane disrupting peptides and a chemical antibiotic seems to be not sufficient for synergism. He et al. (2015) could not find synergistic effects between four different AMPs that were previously characterized in their membrane disrupting capabilities. Nevertheless they confirmed the synergism between magainin II and β-lactamic antibiotics. The methodology used to determine synergy is a major point when discrepancies appear. In fact, the chequerboard experiments which are popularly used as the basis for calculation of a fractional inhibitory concentration index (FICI), is also possibly particularly prone to reproducibility problems (Odds, 2003). In spite of the lack of consensus, the time-kill method could be considered the gold standard for synergism evaluation, as it allows a dynamic evaluation and higher sensitivity, when compared to the other methods.

## Conclusions

Antimicrobial peptides are promising new molecules that can be *de novo* designed in order to meet the challenge of multiresistant bacteria but avoiding host side effects. Understanding the selectivity mechanisms these molecules display to interact with prokaryotic or mammalian membranes would give us the chance to fine-tune the selectivity of an AMP toward the reduction of cytotoxicity and increase antimicrobial activity, even though is a complex task due to the multiple factors affecting lipid selectivity. The particular membrane disruptive activity of AMPs makes them ideal candidates for combined therapies with conventional chemical antibiotics displaying synergistic effects. The recent findings in the field show a promising future for AMPs as enhancers of antimicrobial activity in multidrug resistant bacteria therapies. Nevertheless, more research is needed to overpass their limitations and establish their actual scope.

## Author contributions

AH and MM wrote the manuscript, search for updated bibliography, designed figures and discuss the research topics. PM and LS wrote a section of the manuscript and read and corrected the review. PCM wrote the manuscript, selected the research topics to be addressed, corrected and edit the final version of the review.

### Conflict of interest statement

The authors declare that the research was conducted in the absence of any commercial or financial relationships that could be construed as a potential conflict of interest.
